# miR-620 promotes tumor radioresistance by targeting 15-hydroxyprostaglandin dehydrogenase (HPGD)

**DOI:** 10.18632/oncotarget.4210

**Published:** 2015-06-04

**Authors:** Xiaoyong Huang, Samira Taeb, Sahar Jahangiri, Elina Korpela, Ivan Cadonic, Nancy Yu, Sergey N. Krylov, Emmanouil Fokas, Paul C. Boutros, Stanley K. Liu

**Affiliations:** ^1^ Sunnybrook Research Institute, Sunnybrook Health Sciences Centre, Toronto, Canada; ^2^ Department of Medical Biophysics, University of Toronto, Toronto, Canada; ^3^ Department of Chemistry, York University, Toronto, Canada; ^4^ CRUK/MRC Oxford Institute for Radiation Oncology, Gray Laboratories, Department of Oncology, University of Oxford, Oxford, UK; ^5^ Ontario Institute for Cancer Research, University of Toronto, Toronto, Canada; ^6^ Department of Radiation Oncology, University of Toronto, Toronto, Canada

**Keywords:** miR-620, radiation resistance, HPGD, PGE2

## Abstract

MicroRNA contribute to tumor radiation resistance, which is an important clinical problem, and thus we are interested in identifying and characterizing their function. We demonstrate that miR-620 contributes to radiation resistance in cancer cells by increasing proliferation, and decreasing the G2/M block. We identify the hydroxyprostaglandin dehydrogenase 15-(nicotinamide adenine dinucleotide) (*HPGD/15-PGDH*) tumor suppressor gene as a direct miR-620 target, which results in increased prostaglandin E2 (PGE2) levels. Furthermore, we show that siRNA targeting of HPGD or administration of exogenous PGE2 recapitulates radioresistance. Targeting of the EP2 receptor that responds to PGE2 using pharmacological or genetic approaches, abrogates radioresistance. Tumor xenograft experiments confirm that miR-620 increases proliferation and tumor radioresistance *in vivo*. Regulation of PGE2 levels via targeting of HPGD by miR-620 is an innovative manner by which a microRNA can induce radiation resistance.

## INTRODUCTION

Over 50% of cancer patients will receive radiotherapy as part of their treatment. Despite delivery of a radical course of radiotherapy, tumor recurrences can occur, due to cellular mediators that promote radiation resistance [1], and this, in turn, can result in a more aggressive phenotype including increased proliferative capacity, nodal metastases, and poor prognosis [2–4]. To address this important clinical problem, a better understanding of the molecular mediators of resistance is required. We investigate the role of microRNA (miR) as mediators of tumor aggression and radiation resistance.

miR are short non-coding RNA that bind to the 3′ untranslated (UTR) region of mRNA, resulting in transcript degradation or inhibition of protein translation. A single miR may exhibit pleotropic effects due to regulation of multiple target mRNA. Their expression is known to be dysregulated in malignancies, and they are believed to contribute towards the pathogenesis of cancer [5]. Indeed, they can influence a broad range of cancer-related processes including proliferation, apoptosis, invasion and metastasis. It is now known that miR are involved in the response of tumor cells to radiotherapy (reviewed in [6] and [7]). Frank Slack and colleagues were the first to demonstrate that miR from the let-7 family could directly influence radiosensitivity in *C. elegans* and cancer cells [8]. Dicer and Drosha, which are essential enzymes involved in processing miR, are now known to be involved in activation of the DNA damage response (DDR) [9], further supporting the importance of miR in mediating cellular response to ionizing radiation. miR have been demonstrated to regulate radiosensitivity through targeting essential components of the DDR such as Ataxia-teleangiectasia mutated (ATM) [10], DNA-dependent protein kinase catalytic subunit (DNA-PKcs) [11], histone variant H2AX [12], SNF2H [13], and the p53 (reviewed in [14]), and BRCA1 tumor suppressors [15]. Additionally, miR target critical survival pathways, such as the Akt [16, 17], mitogen-activated protein kinase (MAPK), and sphingosine-phosphate 1 (S1P) signaling pathways [18]. Collectively, this results in alteration of cellular radiosensitivity. However, there are many additional miR that may influence radiosensitivity and these remain to be characterized.

We have now investigated the function of miR-620 in cancer radiation resistance and aggression. Only one paper has investigated the role of miR-620 to date [19]. Zhao et al., recently demonstrated that miR-620 is upregulated in human lung adenocarcinoma, and targets the *Glypican 5* (GPC5) tumor suppressor gene, which alters proliferation, migration and invasion [19]. We now demonstrate that miR-620 overexpression promotes a radioresistant phenotype in a range of cancer cells, increases cellular proliferation and deregulates the G2/M checkpoint following irradiation, and enhances invasiveness. We discovered that miR-620 directly targets the hydroxyprostaglandin dehydrogenase 15-(nicotinamide adenine dinucleotide) (*HPGD/15-PGDH*) tumor suppressor gene. HPGD is a key enzyme that inactivates prostaglandin E2 (PGE2), a proinflammatory lipid which promotes tumorigenesis [20, 21]. We show for the first time that miR-620 downregulation of HPGD promotes radioresistance. Additionally, we demonstrate that PGE2 administration can induce cancer cell radioresistance, and that knockdown of the EP2 receptor or pharmacological inhibition with an EP2 antagonist can abrogate this.

Together, our research provides novel insights into the role of miR-620 in promotion of cancer aggression and radiation resistance, and highlights the relevance of PGE2 in radiation response.

## RESULTS

### miR-620 promotes increased survival, proliferation and G2/M checkpoint deregulation following irradiation

**Figure 1:**
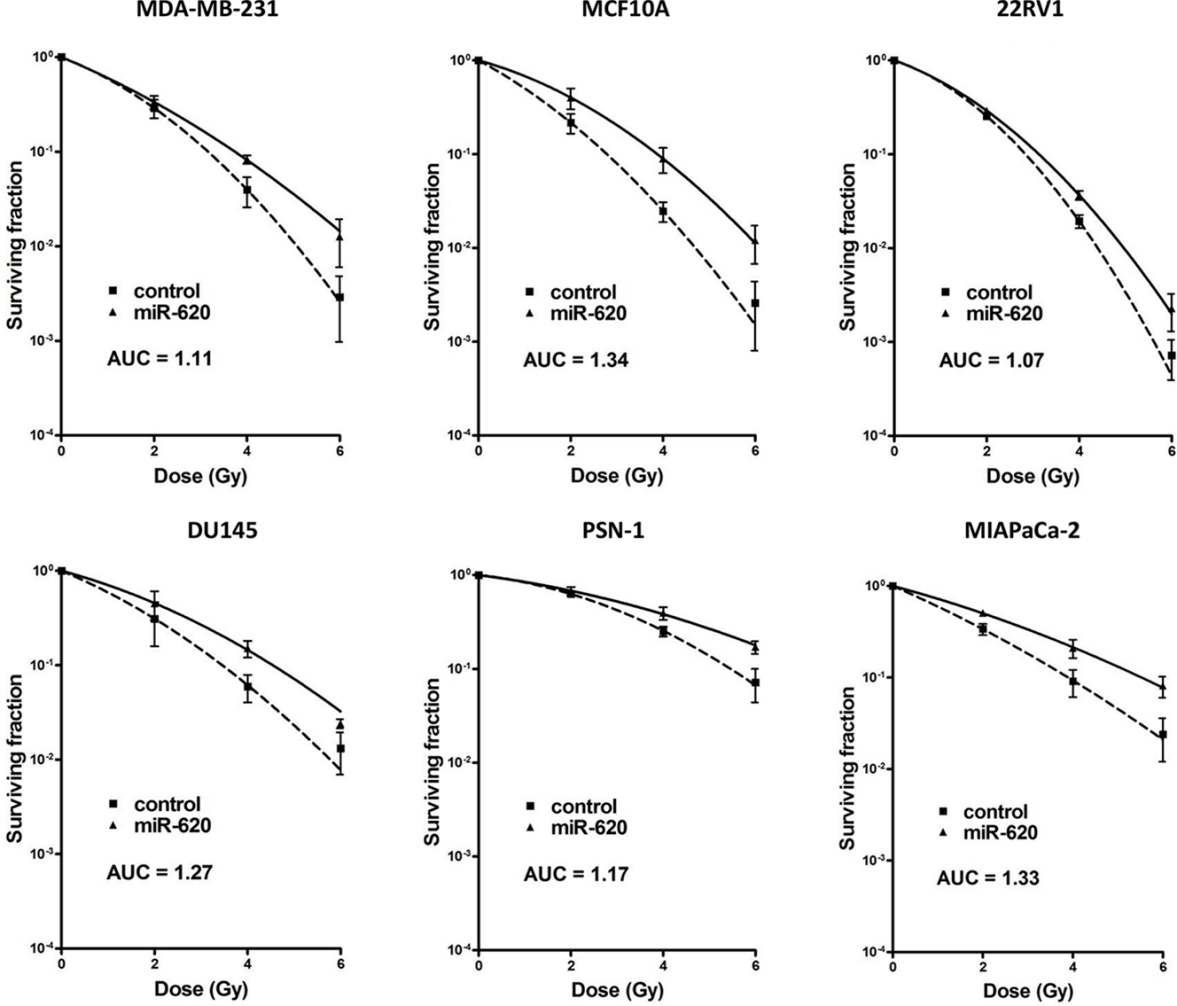
miR-620 promotes radiation resistance MDA-MB-231, MCF10A, DU145, 22RV1, PSN-1 and MIAPaCa-2 cells were transiently transfected with control or miR-620 mimic, radiation clonogenic survival assays performed, and surviving fraction fitted to the linear-quadratic equation. Radiation protection factors (RPF) were determined by dividing the area under the curve (AUC) of the miR-620 mimic by the AUC of the control mimic. There were statistically significant differences in AUC seen for all survival curves (*p* < 0.05).

**Figure 2:**
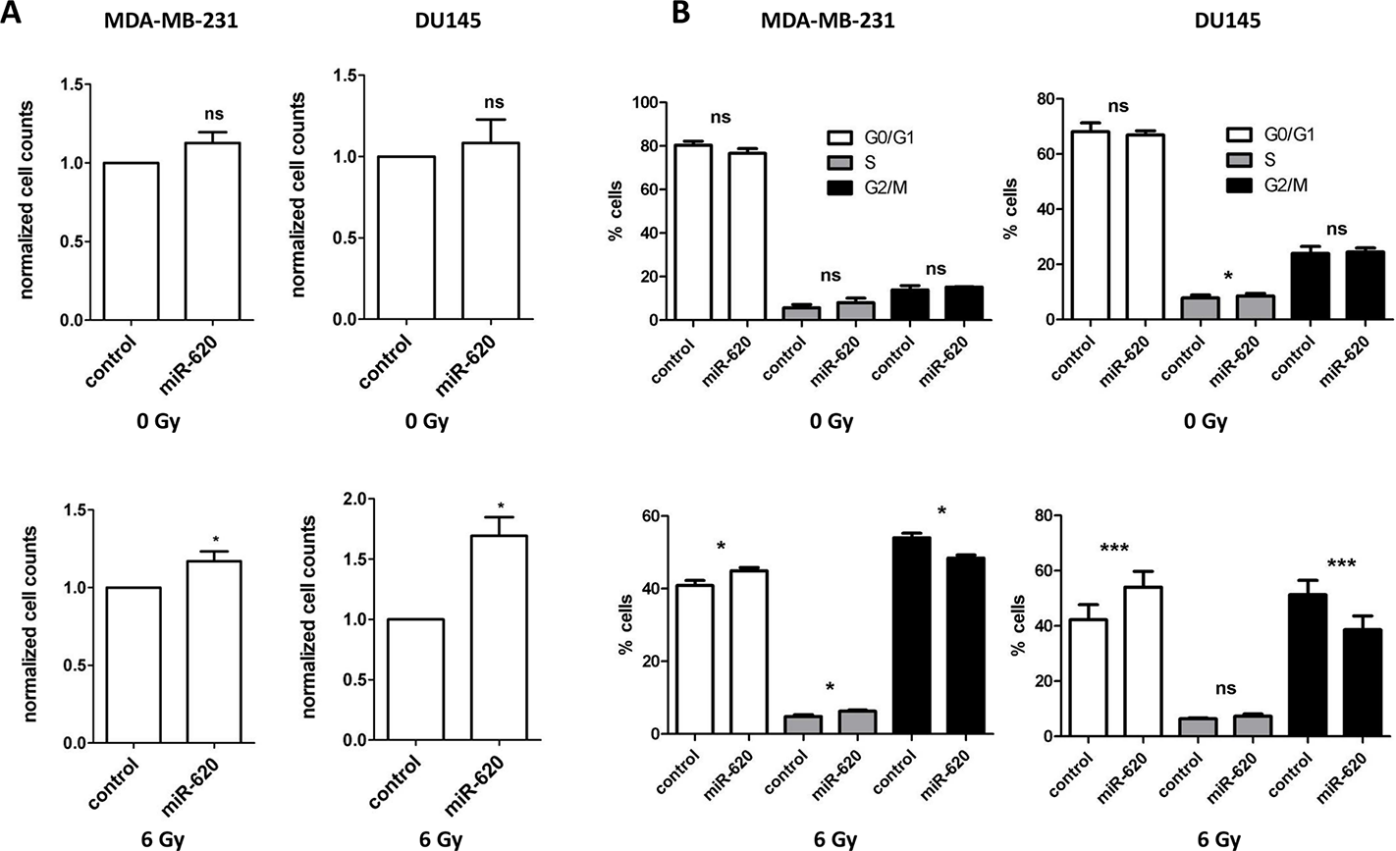
miR-620 increases cellular proliferation and decreases G2/M phase accumulation following irradiation **A.** MDA-MB-231 and DU145 cells were transiently transfected with control or miR-620 mimic, mock irradiated or irradiated with 6 Gy of ionizing radiation, and total viable cells determined after 5 days. **B.** Cell cycle profiles of transiently transfected cells mock irradiated or irradiated with 6 Gy of ionizing radiation. Mean, standard deviations and statistical significance are denoted; *p < 0.05, ***p < 0.01, ns, non-significant difference; *n* = 3 independent experiments.

It is becoming evident that miR play an important role in tumor radiation response. We assessed the influence of a recently characterized miR-miR-620 (described in only one publication to date [19]), on cancer radiation response by transiently transfecting breast, prostate and pancreatic cancer cell lines (MDA-MB-231 breast cancer cells, non-tumorigenic MCF10A breast cells, 22RV1 and DU145 prostate cancer cells, PSN-1 and MIAPaCa-2 pancreatic cancer cells) with a miR-620 mimic or a control mimic and performing radiation clonogenic survival assays. All miR-620 mimic transfected cell lines displayed increased radiation resistance compared to control mimic transfected cells (as indicated with increased radiation protection factors (RPF) in Figure 1), demonstrating that this effect is conserved between different cancer cell types. To determine if this resistance was due to an influence on cellular proliferation, we mock irradiated and irradiated MDA-MB-231 and DU145 cells that were transiently transfected with miR-620 mimic or control mimic and counted viable cells. miR-620 did not significantly alter proliferation of mock irradiated MDA-MB-231 (1.1 ± 0.1 (miR-620) versus 1.0 (control), *p* = ns) or DU145 cells (1.1 ± 0.2 (miR-620) versus 1.0 (control), ns). However, it significantly increased proliferation following a 6 Gy dose of ionizing radiation (IR), relative to control cells (MDA-MB-231: 1.2 ± 0.1 (miR-620) versus 1.0 (control); *p* < 0.05 and DU145: 1.7 ± 0.2 (miR-620) versus 1.0 (control), *p* < 0.05) (Figure 2A). Consistent with this, we discovered that the cell cycle profiles of MDA-MB-231 (G1 phase: 76.6 ± 2.2% (miR-620) versus 80.3% ± 3.2% (control), ns; S phase: 8.1 ± 3.6% (miR-620) versus 5.7 ± 2.5% (control), ns; G2/M phase: 15.2 ± 0.2% (miR-620) versus 13.8 ± 2.0% (control), ns) and DU145 cells (G1 phase: 66.9 ± 1.5% (miR-620) versus 68.1 ± 3.1% (control), ns; S phase: 8.6 ± 1.6% (miR-620) versus 7.8 ± 1.8% (control), ns; G2/M phase: 24.5 ± 2.6% (miR-620) versus 23.9 ± 2.5% (control), ns) were not altered by miR-620 mimic in mock irradiated cells. However, 24 h after IR, the control MDA-MB-231 (G2/M phase: 48.4 ± 1.4% (miR-620) versus 54.0 ± 2.2% (control), *p* < 0.05) and DU145 cells (G2/M phase: 38.6 ± 8.6% (miR-620) versus 51.3 ± 8.9% (control), *p* < 0.01) demonstrated an accumulation of cells in G2/M, which was significantly less in miR-620 transfected cells (Figure 2B). The extent of G2/M deregulation was lower in MDA-MB-231 cells compared to DU145 cells, however. Thus, increased expression of miR-620 induces radioresistance, increases proliferative capacity and deregulation of the G2/M checkpoint following irradiation.

### miR-620 increases cellular invasiveness

**Figure 3:**
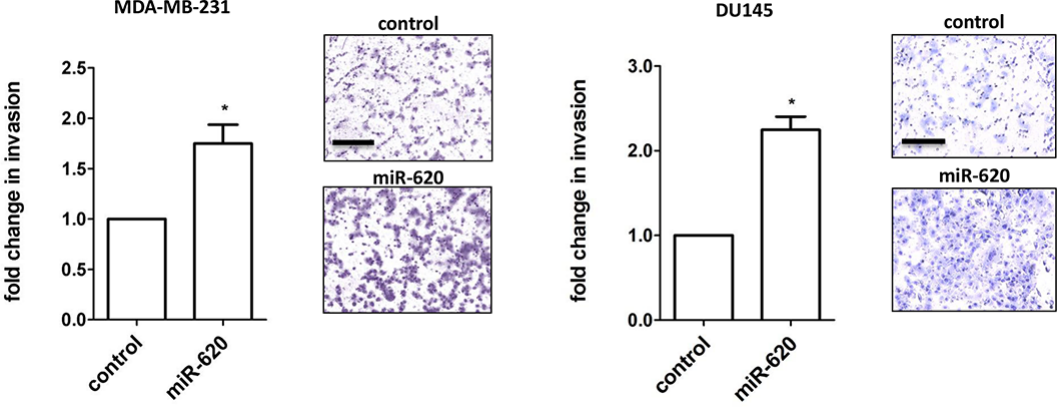
miR-620 increases invasiveness Invasion assays were performed on MDA-MB-231 and DU145 cells transiently transfected with control or miR-620 mimic. Mean, standard deviations and statistical significance are denoted; *p < 0.05; *n* = 3 independent experiments. Representative images are shown; scale bar = 250 μm.

Increased invasiveness may promote metastatic spread, and thus we assessed the influence of miR-620 on invasion using the Matrigel transwell assay. miR-620 overexpression significantly increased the invasiveness of MDA-MB-231 and DU145 cells (1.7 ± 0.2 (miR-620) versus 1.0 (control); *p* < 0.05) and DU145 cells (2.2 ± 0.15 (miR-620) versus 1.0 (control); *p* < 0.05) (Figure 3). Collectively, miR-620 can promote an aggressive phenotype in both MDA-MB-231 and DU145 cells by increasing survival and proliferation following radiation treatment, and enhancing invasive capacity.

### HPGD is a target of miR-620 and mediates radiation resistance

To identify downstream effectors of miR-620 potentially mediating radioresistance, we performed *in silico* target prediction using Targetscan Human release 6.0 [22]. Targetscan identified the tumor suppressor gene, hydroxyprostaglandin dehydrogenase 15-(nicotinamide adenine dinucleotide) (*HPGD/15-PGDH*) as a putative target of miR-620. HPGD is the key enzyme that inactivates a number of bioactive lipids, and is also a tumor suppressor [20]; its expression is reduced in a range of human cancers relative to corresponding normal tissue [23, 24, 25]. HPGD catalyzes the degradation of prostaglandin E2 (PGE2), a bioactive eicosanoid that is associated with tumor progression [26, 27]. Its substrate, PGE2, has been previously demonstrated to provide radioprotection for normal intestinal stem cells [28–30]. Thus, we were interested in determining whether miR-620 targets HPGD, thereby increasing levels of PGE2 to promote radioresistance.

**Figure 4:**
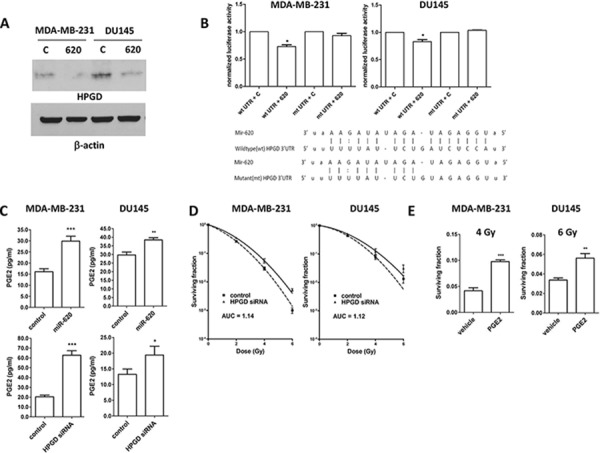
HPGD is a target of miR-620, reduces cellular PGE-2 levels and induces radiation resistance **A.** Representative western blot for HPGD and β-actin (loading control) levels in MDA-MB-231 and DU-145 cells transiently transfected with control (C) or miR-620 (620) mimic. **B.** MDA-MB-231 and DU145 cells were transiently cotransfected with wildtype (wt) or mutant (mt) HPGD 3′UTR luciferase vector, renilla vector and control or miR-620 mimic, then firefly luciferase assay performed and normalized to renilla signal. Sequences of the miR-620 binding sequence in the HPGD wt and mt 3′UTR are shown. **C.** MDA-MB-231 and DU145 cells transiently transfected with control or miR-620 mimic, or transiently transfected with control or HPGD siRNA were lysed and subjected to PGE2 assays (representative experiments shown). **D.** MDA-MB-231 and DU145 cells were transiently transfected with control or HPGD siRNA and radiation clonogenic survival assays performed. **E.** MDA-MB-231 and DU145 cells were treated with PGE2 or vehicle, irradiated (4 Gy or 6 Gy, respectively) and radiation clonogenic survival assays performed. Mean, standard deviations and statistical significance are denoted; *p < 0.05, **p < 0.01, ***p < 0.001; *n* = 3 independent experiments.

Western blotting of lysates from MDA-MB-231 and DU145 cells transfected with control or miR-620 mimic established that HPGD was decreased at the protein level (Figure 4A). We co-transfected a luciferase reporter vector bearing the wildtype 3′UTR of HPGD into MDA-MB-231 or DU145 cells with control or miR-620 mimic. The presence of miR-620 resulted in a significant reduction in normalized luciferase units relative to control (MDA-MB-231: 0.72 ± 0.03, *p* < 0.05; DU145: 0.83 ± 0.03, *p* < 0.05) (Figure 4B). However, mutation of the predicted miR-620 binding site reconstituted luciferase activity with no significant difference relative to control (MDA-MB-231: 0.92 ± 0.04; DU145: 1.03 ± 0.01) (Figure 4B).

**Figure 5:**
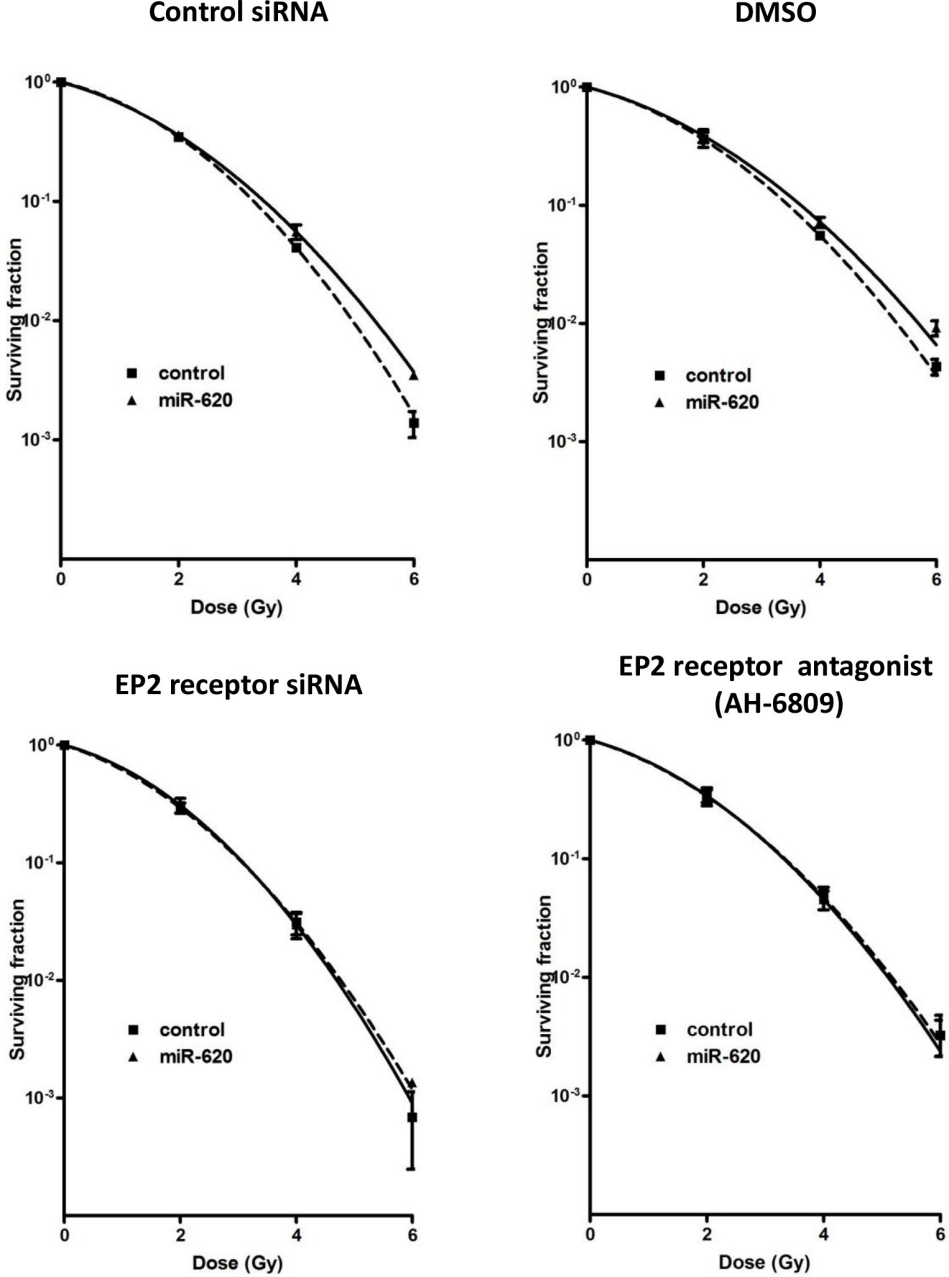
EP2 receptor is responsible for miR-620-mediated radioresistance MDA-MB-231 cells stably expressing control miR or miR-620 were transiently transfected with control or EP2 receptor siRNA (left panel), or were treated with vehicle (DMSO) or the EP2 antagonist AH-6809 (right panel), and radiation clonogenic survival assays performed. Mean and standard deviations are denoted; *n* = 3 independent experiments.

We next assayed cellular PGE2 levels (the key substrate for HPGD), and discovered that miR-620 mimic transfected cells have higher levels of PGE2 compared to control cells (MDA-MB-231: 29.8 ± 2.1 (miR-620) versus 16.0 ± 1.3 (control); *p* < 0.001; DU145: 38.4 ± 1.2 (miR-620) versus 29.7 ± 1.6 (control); *p* < 0.01) (Figure 4C). Similarly, knockdown of HPGD with siRNA increased PGE2 levels (MDA-MB-231: 62.7 ± 4.6 (HPGD siRNA) versus 20.4 ± 1.7 (control); *p* < 0.001; DU145: 19.4 ± 2.8 (HPGD siRNA) versus 13.2 ± 1.6 (control); *p* < 0.05) (Figure 4C). Knockdown of HPGD increased radiation resistance in both MDA-MB-231 and DU145 cells (Figure 4D); reduction of HPGD protein levels were confirmed by western blotting (Supplementary Figure 1). For MDA-MB-231 cells, RPFs were similar between HPGD knockdown compared to miR-620 overexpression. However, for DU145 cells, HPGD knockdown resulted in a slightly lower RPF compared to miR-620 overexpression. Together, this demonstrated that HPGD knockdown could phenocopy the radioresistance seen in miR-620 overexpressing cells. Similarly, treatment with exogenous PGE2 rendered cells more resistant to radiation (MDA-MB-231: 0.097 ± 0.003 (PGE2) versus 0.041 ± 0.003 (vehicle); *p* < 0.001; DU145: 0.056 ± 0.004 (PGE2) versus 0.033 ± 0.002 (vehicle); *p* < 0.01) (Figure 4E). PGE2 signaling occurs through E Prostanoid (EP) receptors, and the EP2 receptor has been demonstrated to mediate radiosensitivity of normal small intestinal crypt cells [30]. Thus to determine whether the EP2 receptor was responsible for the observed radiation resistance induced by miR-620, we used siRNA to knockdown the EP2 receptor (Supplementary Figure 2) and performed radiation clonogenic survival assays in MDA-MB-231 cells stably overexpressing miR-620 or control miR (Figure 5). Knockdown of the EP2 receptor promoted cancer cell radiosensitivity to a greater degree in the miR-620 overexpressing cells relative to control cells. Similarly, pharmacological antagonism of the EP2 receptor with AH-6809 was able to radiosensitize MDA-MB-231 cells stably overexpressing control and miR-620, with greater sensitization seen again in the miR-620 overexpressing cells (Figure 5). This indicated that the EP2 receptor is an important intermediary for miR-620-mediated radiation resistance.

### miR-620 promotes tumor radiation resistance *in vivo*

**Figure 6:**
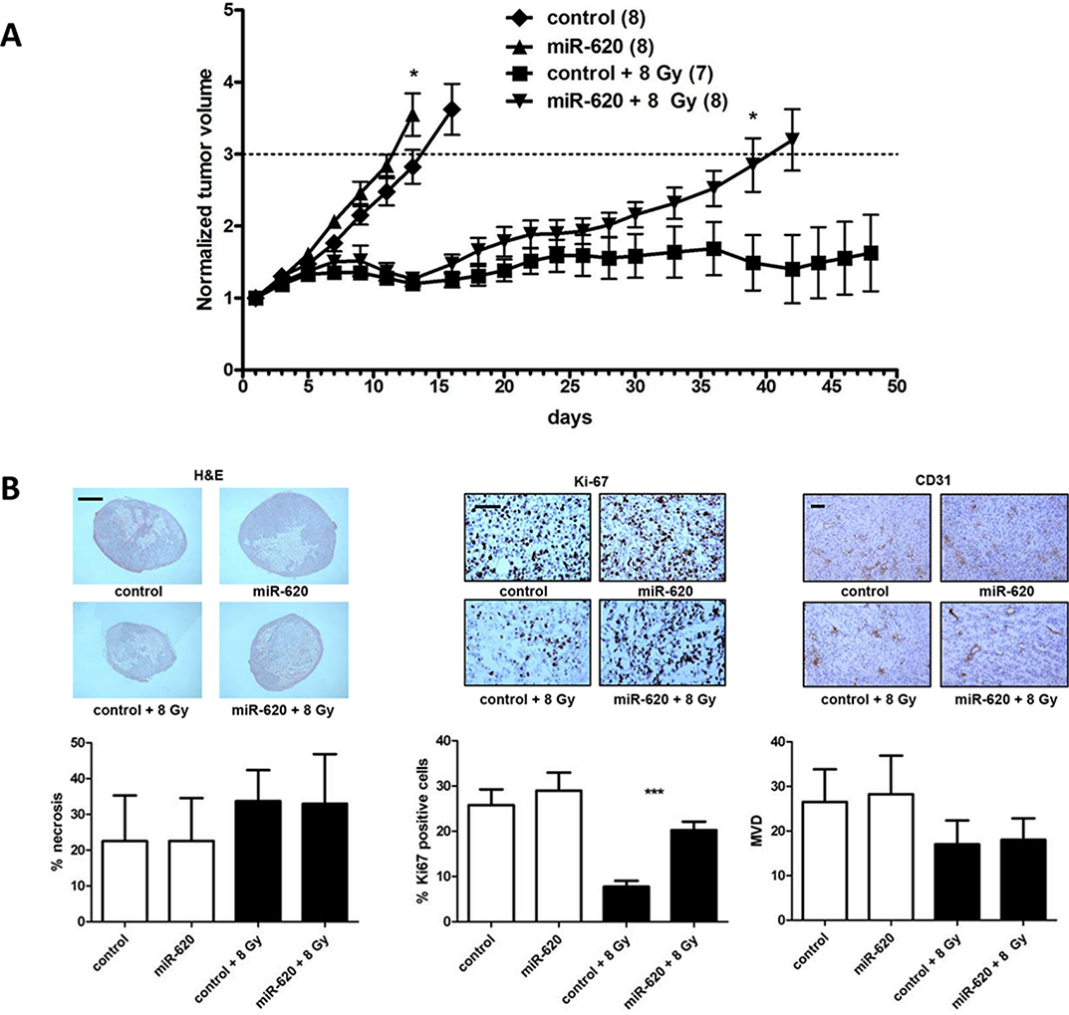
miR-620 promotes tumor radioresistance *in vivo* **A.** DU145 cells stably overexpressing control or miR-620 were grown as subcutaneous tumors, mock irradiated or irradiated with 8 Gy of ionizing radiation, and tumor volumes measured. **B.** Tumors were stained with H&E or immunostained with anti-ki67 or anti-CD31 antibodies, and necrosis, proliferation and mean vessel density (MVD) quantitated respectively. Scale bar indicates 2 mm (H&E) or 200 μm. Mean, standard deviations and statistical significance are denoted; *p < 0.05, ***p < 0.001.

To investigate the *in vivo* scenario of miR-620 overexpression in tumors on growth and radiation growth delay, we used DU145 cells stably overexpressing miR-620 or control miR to produce subcutaneous tumors in athymic nude mice, followed by irradiation of the tumors with an 8 Gy dose of IR or mock IR (Figure 6A). The mock irradiated DU145-miR-620 tumors grew slightly more quickly than the DU145-control tumors (tumor volumes were significantly larger based upon day 13 values, *p* < 0.05). A tumor growth delay was observed in both sets of tumors following irradiation, however the DU145-miR-620 tumors began to regrow by day 16, whereas the DU145-control tumors did not continue to grow following irradiation (significant difference in tumor volumes based upon day 39 values, *p* < 0.05) (Figure 6A). This provided *in vivo* evidence that miR-620 overexpression renders tumor radioresistant.

Tumors were removed and haemotoxylin and eosin staining was performed to identify regions of tumor necrosis. Irradiation increased tumor necrosis compared to unirradiated tumors, however there was no significant difference in percentage of necrosis visualized between DU145-miR-620 tumors and DU145-control tumors (32.94 ± 13.8% (miR-620) versus 33.7 ± 8.6% (control); *p* = ns) (Figure 6B). Ki-67 immunostaining indicated that the irradiated DU145-miR-620 tumors contained a significantly greater percentage of proliferative cells compared to control tumors (20.2 ± 1.8% (miR-620) versus 7.7 ± 1.2% (control); *p* < 0.001). In unirradiated tumors, there was no significant difference in proliferative cells (28.9 ± 3.9% (miR-620) versus 25.7 ± 3.4% (control); *p* = ns) (Figure 6B). Since miR are noted to influence angiogenesis, we performed anti-CD31 immunostaining followed by microvessel density (MVD) determination. MVD was not significantly altered in the DU145-miR-620 tumors compared to the DU145-control tumors that were not irradiated (28.2 ± 8.6 (miR-620) versus 26.5 ± 7.3 (control); *p* = ns) or irradiated (18.0 ± 4.8 (miR-620) versus 17.0 ± 5.3 (control); *p* = ns) (Figure 6B). Together, the immunohistochemistry data support our observations that miR-620 overexpression in DU145 miR-620 tumors promotes radiation resistance *in vivo* by increasing proliferation.

## DISCUSSION

miR are an integral contributor to tumor radiation response, and thus elucidation of their function is essential to understanding this clinical problem. We provide novel evidence that miR-620 can promote radiation resistance in a range of cancer cells, including those that are inherently more radioresistant (i.e., pancreatic cancer). Our research provides the first link between miR regulation of PGE2 and radiation resistance, which occurs through alteration of PGE2 levels via targeting of HPGD.

PGE2 can induce pleotropic tumorigenic effects that include enhanced proliferation, migration, invasion, and angiogenesis [31–33]. Signaling by PGE2 occurs through four EP receptors, and their expression levels are altered in cancer [34]. Additionally, since they are coupled to different signaling pathways, their phenotypic outcome (e.g., tumor progression or inhibition) can differ. Expression of EP2 and EP4 receptors are elevated in prostate cancer specimens compared to non-malignant prostate [35, 36]. EP4 expression increases during progression of prostate cancer from an androgen-sensitive state to castrate-resistance [37], highlighting the distinct phenotypic effects that these receptors can possess. The use of aspirin (which inhibits cyclo-oxygenase 1 (COX-1) and 2 (COX-2) production) has been associated with a reduction in prostate cancer specific mortality in patients treated with radiotherapy or radical prostatectomy [38]. Mechanistically, it has been demonstrated that the COX-2 inhibitor, celecoxib, induces prostate cancer death through the EP2 receptor [39]. In a murine model of cyclo-oxygenase 2 (COX-2) driven mammary cancer, the EP1, EP2, and EP4 receptors were upregulated in tumor tissue [40]. Treatment of these tumors with celecoxib reduced tumor growth and reduced microvessel density [40]. Similarly, Tian and Schiemann observed that loss of EP2 receptor reduced breast cancer growth, angiogenesis, and metastases in an orthotopic mouse model [41].

Accumulating evidence in the literature supports the overarching view that mechanisms resulting in increased production and signaling from PGE2 promotes tumor therapy resistance. Kurtova *et al*. recently reported that chemotherapy can induce PGE2 release in bladder cancer, and that this promotes cancer stem cell (CSC) repopulation and treatment resistance [42]. They noted that the CSC repopulation was blocked by a PGE2-neutralizing antibody and celecoxib-mediated blockade of PGE2 signalling. Although we have not specifically assayed for miR-620 and HPGD effects on CSC frequency in our cancer cell models, it is clear that miR-620 overexpression, HPGD knockdown or PGE2 treatment can increase clonogenic survival following irradiation. Clonogenic survival is the gold standard assay for irradiation studies, and takes into account all modes of cell death following irradiation including mitotic catastrophe, terminal senescence, apoptosis and presumably the influence of CSC repopulation. The Li laboratory demonstrated that in irradiated tumors, activation of caspase 3 by apoptotic tumor cells induces PGE2, which can stimulate the growth of surviving tumor cells to repopulate the tumor [43]. PGE2 appears to have a conserved effect in radiation response, since it increases hematopoietic stem cell survival after total body irradiation [44].

**Figure 7:**
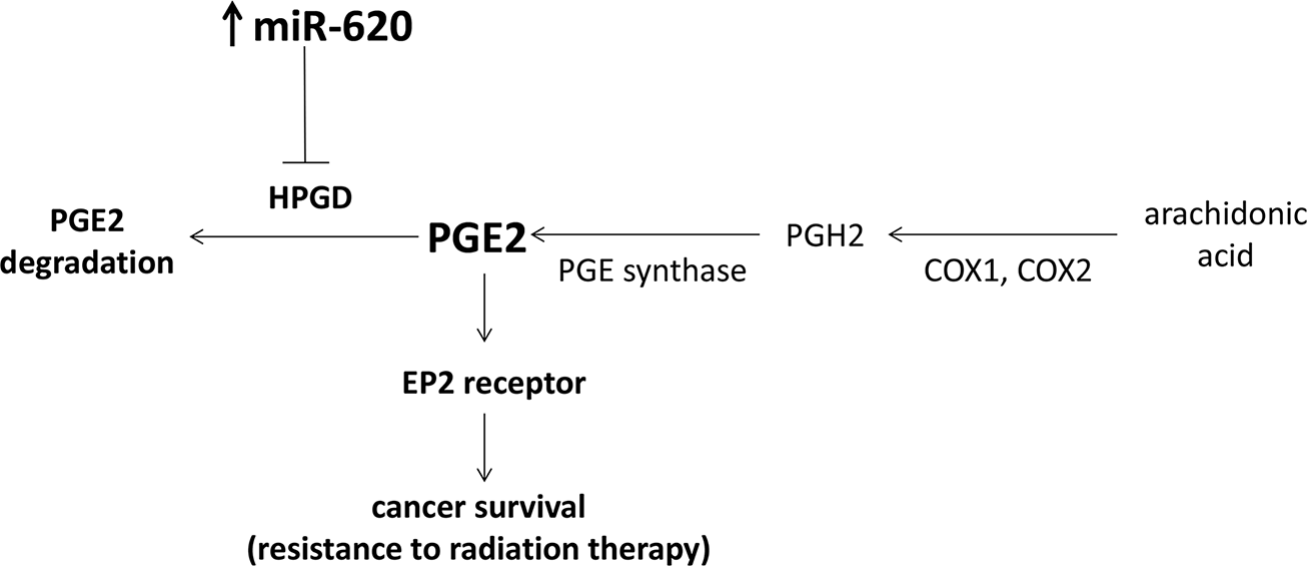
Proposed model of miR-620 on cancer survival in response to radiation treatment PGE2 is synthesized through the action of COX1 and COX2 on arachidonic acid, followed by PGE synthase. This is counteracted by degradation of PGE2 by its major catabolizing enzyme, HPGD. miR-620 specifically targets HPGD for degradation which results in increased PGE2 levels, and signaling through the EP2 receptor promotes cancer cell survival.

PGE2 has been shown to promote prostate cancer proliferation [39], and angiogenesis (via VEGF secretion) and this also appears to be mediated primarily by the EP2 receptor [32, 33]. We have now demonstrated that the EP2 receptor is also important for promotion of radiation resistance by miR-620, as its genetic loss or pharmacological inhibition can radiosensitize cancer cells that overexpress miR-620. Thus, our research adds to the importance of the EP2 receptor in tumor progression and aggression, by demonstrating its role in promotion of cancer cell survival following irradiation. Together, our results provide support for a model whereby miR-620 targets HPGD, resulting in accumulation of HPGD's substrate, PGE2, and signaling by PGE2 through the EP2 receptor results in cancer radiation resistance (Figure 7). Mohamed *et al*. demonstrated that elevated levels of the TMPRSS2-ERG fusion protein found in prostate cancer, can decrease HPGD expression by binding to the HPGD promoter, resulting in PGE2-dependent cancer proliferation and invasion [45]. However, epigenetic mechanisms also play an important role in regulation of HPGD expression. Thiel et al., discovered that HPGD expression is downregulated in gastric cancer, and provided evidence that this occurred partly through promoter methylation [24]. Our findings indicate that miR-targeting is another epigenetic mechanism that can regulate HPGD expression. Indeed, Lu and colleagues also recently demonstrated that HPGD is decreased by miR-21, and that increased PGE2 promotes cholangiocarcinoma growth [46]. In addition to regulation of PGE2 degradation by HPGD, it is known that the generation of PGE2 is also tightly regulated.

COX-2 is an inducible enzyme that catalyzes the synthesis of prostaglandins from arachindonic acid, and is a rate-limiting enzyme in PGE2 production. Not surprisingly, COX-2 induction is associated with increased production of PGE2 [47]. Analysis of data from the Radiation Therapy Oncology Group (RTOG)-9202 trial which randomized men with prostate cancer to short or long term androgen-deprivation therapy, demonstrated that COX-2 expression was associated with biochemical failure and distant metastasis [48]. A more recent analysis of this trial data indicated that high expression of COX-2 in prostate cancer, in combination with three other genes (*ki-67*, *MDM2* and *p16*), predicted for an increased risk of developing distant metastases following androgen deprivation therapy and radiotherapy [49]. In addition to its utility as a prognostic biomarker, research has also investigated COX-2 as a therapeutic target [50]. Multiple preclinical studies have demonstrated proof-of-principle of COX-2 inhibition for enhancement of tumor radiation response (reviewed in [51]). Several early phase trials are investigating the COX-2 inhibitor, celecoxib, as a radiosensitizer in a range of malignancies including prostate cancer [52], locally advanced non-small cell lung cancer [53], locally advanced cervical cancer [54], rectal cancer, and recurrent head and neck cancer [55]. The acute toxicities have generally been well-tolerated, although Herrera et al. reported higher than expected late complications [54]. COX-2 inhibitors are also known to increase the risk of cardiovascular events in patients [56], and thus this may limit their utility as tumor radiosensitizers. We demonstrated that specific blockade of the EP2 receptor has therapeutic efficacy in reversing radioresistance, and thus the use of specific inhibitors of PGE2 signaling such as EP2 antagonists may be desirable, since they may potentially limit or avoid side-effects seen with COX-2 inhibitors. Nevertheless, future investigations are needed to establish the potential radiosensitization of critical normal tissues (e.g., small intestine, spinal cord) by EP2 antagonists, and thereby allow determination of the therapeutic ratio.

The post-transcriptional regulation of COX-2 by different miR has been well described by multiple research groups (reviewed in [57]). In this context, these miR would presumably exhibit a tumor inhibiting function by downregulating PGE2 production. Our research adds a new element of complexity, by demonstrating for the first time, that miR-620 can target the major PGE2 catabolizing enzyme, HPGD, and promote tumor radiation resistance. Additionally, we provide evidence that pharmacological antagonism of the EP2 receptor may be a useful therapeutic strategy to enhance tumor radiation response.

## MATERIALS AND METHODS

### Cell lines and cell culture

Human prostate adenocarcinoma (DU145, 22RV1), human breast adenocarcinoma (MDA-MB-231) and human pancreatic adenocarcinoma (MIAPaCa-2, PSN-1) cell lines were purchased from American Type Culture Collection (ATCC; VA, USA). Early passage cell lines were cultured in Dulbecco's modified Eagle medium (DMEM) with the exception of PSN-1 which were cultured in RPMI-1640 medium, containing 4.5 g/L glucose (Invitrogen, Ontario, Canada) supplemented with 10% fetal bovine serum (FBS) (Invitrogen, Ontario, Canada) and penicillin (100 U/mL) – streptomycin (100 μg/mL) (Invitrogen, Ontario, Canada) (hereafter referred to as 10% DMEM), and maintained in a humidified 37°C incubator with 5% CO_2_. MCF10A immortalized human mammary epithelial cells were kindly provided by Dr. Senthil Muthuswamy, and cultured in DMEM/F12 supplemented with 5% horse serum (Invitrogen, Ontario, Canada), epidermal growth factor (EGF) (20 ng/mL), (Sigma-Aldrich, Ontario, Canada) hydrocortisone (0.5 mg/mL) (Sigma-Aldrich, Ontario, Canada), cholera toxin (100 ng/mL) (Sigma-Aldrich, Ontario, Canada), insulin (10 ug/mL) (Sigma-Aldrich, Ontario, Canada) and penicillin-streptomycin. Cell lines were passaged when they reached approximately 80% confluency and were regularly tested with MycoAlert (Lonza, Ontario, Canada) to ensure the absence of mycoplasma contamination.

### Transfection of microRNA mimics and siRNA

3 × 10^5^ cells were seeded into 6-well plates, then 16 h later, miScript miRNA miR-620 mimic or miRNA control mimic (Thermo Fisher Scientific, MA, USA) were mixed with 6 μL of DharmaFECT transfection reagent (Thermo Fisher Scientific, MA, USA) and DMEM as per the manufacturer's instructions, then added to 10% DMEM for transfection of the cells. For siRNA transfections, control and siRNA for HPGD or EP2 receptor (Santa Cruz Biotechnology, CA, USA) were transiently transfected into cells using Lipofectamine 2000 (Invitrogen, Ontario, Canada) as per manufacturer's recommendations, and 24 h later, radiation clonogenic survival assays (described below) were performed on the transfected cells.

### EP2 receptor antagonist

Vehicle (DMSO), or EP2 antagonist (AH-6809; Santa Cruz Biotechnology, USA) was added at 40 μM to cells for 3 hrs prior to irradiation.

### Generation of stable overexpressing miR-620 cell lines

Cells were transduced with shMIMIC miR-620 or non-silencing control lentiviral particles as per manufacturer's instructions (ThermoScientific, PA, USA), selected using puromycin for two weeks, and stable transductants were pooled.

### Cellular proliferation assay

24 h after transient transfection of cells with miR-620 or control mimic, cells were seeded in triplicate in 10% DMEM in 6-well plates and mock irradiated or irradiated with a 6 Gy dose of IR 6 hr later. Four days later, cells were trypsinized and total viable cell number determined using the Countess automated cell counter (Life Technologies, Ontario, Canada); cell numbers were normalized relative to control cells.

### Cell cycle analysis

Cells were mock irradiated or irradiated with a 6 Gy dose of IR, then 24 h later, cells were trypsinized, washed in PBS and fixed in ice-cold 80% ethanol in Hank's Buffered Salt Solution (HBSS) (137 mM NaCl, 5.4 mM KCl, 0.25 mM Na_2_HPO_4_, 0.44 mM KH_2_PO_4_, 1.3 mM CaCl_2_, 1.0 mM MgSO_4_, 4.2 mM NaHCO_3_) for 30 min on ice. Fixed cells were collected by centrifugation, washed twice with PBS, and resuspended in 50 μg/mL propidium iodide (Sigma-Aldrich, Ontario, Canada) with 0.6% NP-40 (Thermo Fisher Scientific, IL, USA) and 0.1 mg/mL RNAse A in HBSS for 30 min at room temperature in the dark. Cells were then collected by centrifugation, resuspended in PBS, and 20, 000 events captured on a FACSCalibur flow cytometer (BD Biosciences, Ontario, Canada) and cell cycle profile analyzed using FlowJo 10.0.4 (Tree Star Inc., OR, USA).

### Radiation clonogenic survival assay

Cells were seeded onto a six-well plate in 10% DMEM in triplicate and mock irradiated (0 Gy) or irradiated with 2, 4, or 6 Gy dose of IR, respectively. Then cells were placed in a humidified CO_2_ incubator at 37°C to allow colonies to form. Colonies were stained with crystal violet staining solution (0.5% crystal violet (Sigma-Aldrich, Ontario, Canada), 25% methanol) and counted. Survival was expressed as the relative plating efficiencies of the treated cells compared with that of the mock-irradiated cells. The experiments were performed three separate times. Radiation dose-response curves were created by fitting the data to the linear-quadratic equation *S* = e^−*αD*^−βD2^^ using GraphPad Prism 5.0 (GraphPad Software Inc, CA, USA), where *S* is the surviving fraction, *α* and *β* are inactivation constants, and *D* is the dose in Gy. The area under the curves (AUC) which represent the mean inactivation dose (MID) were also calculated using GraphPad Prism. The radiation protection factor (RPF) was calculated by dividing the MID of the test cells by the MID of control cells.

### Real-time quantitative PCR

For microRNA expression, total microRNA was extracted from cells or tumors using the mirVana miRNA kit (Invitrogen, Ontario, Canada) according to the manufacturer's instructions. cDNA was synthesized using the miScript II RT kit (Qiagen, Ontario, Canada) as per manufacturer's instructions. The mature miR-620 expression level was quantified through quantitative real-time PCR using the mi-Script SYBR Green PCR kit (Qiagen, Ontario, Canada) and miScript Primer Assay for SNORD61 and miR-620 (Qiagen, Ontario, Canada) on the StepOnePlus Real-time PCR system (Life Technologies, Ontario, Canada). For gene expression, RNA was extracted using the RNeasy Mini kit (Qiagen, Ontario, Canada) and cDNA synthesized using Omniscript RT kit (Qiagen, Ontario, Canada) as per manufacturer's instructions. HPGD expression level was quantified through quantitative real-time PCR using the QuantiTect SYBR Green PCR kit (Qiagen, Ontario, Canada) on the StepOnePlus Real-time PCR system. For both microRNA and mRNA, expression levels were calculated using the comparative Ct method via StepOne Software (Life Technologies, Ontario, Canada), and relative expression levels normalized to SNORD61 (for microRNA) or GAPDH (for mRNA)). Primer sequences: HPGD: forward 5′-TGCTTCAAAGCATGGCATAG-3′, reverse 5′-AACAAAGCCTGGACAAATGG-3′.

### Matrigel transwell invasion assay

Cells were serum starved overnight (0.1% DMEM), then 2 × 10^5^ cells were seeded on top of 8 μm transwell inserts (BD Biosciences, Ontario, Canada) with 0.1% DMEM and pre-coated with Matrigel (Becton, Dickinson and Company, Ontario, Canada); 10% DMEM was used as a chemoattractant. After 24 h, cells that had invaded through the Matrigel coated transwell inserts were fixed, stained by Kwik-Diff Stain (Thermo Fisher Scientific, Ontario, Canada) and number of invading cells counted under 10 × using a Leica DM LB2 microscope (Leica Microsystems, Ontario, Canada).

### Western blotting

Cells were lysed in ice-cold radioimmunoassay precipitation assay (RIPA) lysis buffer (50 mM Tris pH 7.5, 150 mM NaCl, 2 mM EDTA pH 8.0, 0.5% (v/v) Triton X-100, and Complete protease inhibitor cocktail (Roche, Quebec, Canada)). Cell debris and insoluble material were removed by centrifugation at 12, 000 g at 4°C for 20 min. Following protein quantitation using the Bradford protein assay (Bio-Rad, Ontario, Canada), 25 μg of lysate was loaded per lane and proteins resolved by 4%–20% gradient SDS-PAGE gel, wet-transferred to polyvinylidene fluoride membranes (EMD Millipore, MA, USA), and the membranes were incubated in 5% nonfat dry milk in Tris-buffered saline Tween-20 (TBST) (10 mM Tris-Base, 150 mM NaCl, 0.05% Tween-20; pH 7.4) for 1 h at room temperature to block nonspecific antibody binding, followed by incubation with primary antibody in 5% milk in TBST overnight at 4°C with gentle agitation. The membranes were washed three times for 10 min each in TBST, then incubated in TBST at room temperature for 1 h, followed by three 10-min washes with TBST. Protein-antibody binding on the membranes was detected with the use of enhanced chemiluminescence (ECL) Plus solution (GE Healthcare Life Sciences, Quebec, Canada) followed by exposure of the membranes to x-ray film (FujiFilm, Ontario, Canada). Anti-HPGD antibody was purchased from Sino Biological, China. Anti-β-actin antibody was purchased from Santa Cruz Biotechnology, CA, USA.

### Luciferase assay

For HPGD 3′UTR luciferase assays, cells were transiently co-transfected with a HPGD 3′UTR luciferase reporter plasmid or HPGD 3′UTR luciferase reporter plasmid with mutations in the predicted miR-620 binding site, pcDNA3 vector constitutively expressing Renilla luciferase, and miScript miRNA miR-620 mimic or control mimic. 24 h later, cells were processed for firefly luciferase and Renilla luciferase activity using the Dual Glo Luciferase Assay System (Promega, WI, USA). To normalize for transfection efficiency, the firefly luciferase activity was normalized to the Renilla luciferase activity.

### PGE-2 assay

Cells were lysed in homogenization buffer, lysates sonicated, and processed through a QIA Shredder (Qiagen, Ontario, Canada). Lysate was then clarified by centrifugation, total protein concentrations determined, and PGE2 levels assayed using the Prosaglandin E2 EIA kit (Cayman Chemical Company, Michiagan, USA) as per manufacturer's recommendations.

### Tumor xenograft experiments

All experiments involving mice were performed according to University of Toronto and Sunnybrook Research Institute guidelines, using a peer-reviewed animal protocol. Three million DU145-control and DU145-miR-620 cells were mixed in a 1:1 (vol:vol) ratio with Growth Factor Reduced Matrigel (Becton, Dickinson and Company, Ontario, Canada), and the mixture was injected subcutaneously into the right flanks of 6 to 7-week-old female athymic nude mice (Harlan, Ontario, Canada). Tumor volume (in mm^3^) was determined by caliper measurements performed every 3 to 4 days and calculated by using the modified ellipse formula (volume = length × width^2^/2). When the xenograft tumor volumes reached approximately 100 mm^3^, mice were randomly assigned to mock IR or an 8 Gy dose of IR delivered to the tumor, and tumor volumes determined every 3–4 days after IR. When tumor volumes reached three times the starting volume (except for DU145-control irradiated tumors; these tumors were harvested at day 48), the mice were killed by cervical dislocation, and their tumors were excised, cut in half, with one half placed in Tissue-Tek O.C.T. compound (Fisher Scientific Co., Ottawa, Ontario) and stored at −80°C until cryosectioning and the remaining half flash-frozen in liquid nitrogen.

### Immunohistochemistry

To quantitate tumor necrosis, cellular proliferation and angiogenesis, five micron thick tumor sections were stained with haematoxylin and eosin (H&E), anti-Ki-67 or anti-CD31 antibodies respectively (*n* = 3 tumors per group) as previously described [58]. Areas of necrosis were delineated, quantitated and expressed as a percentage of total tumor area. The percentage of Ki-67 positive nuclei were quantitated from 6 representative fields. Microvessel density (MVD) was determined by finding tumor areas with the highest vascularity (hot spots) on low magnification (5×), and in each hot spot, CD31 positive microvessels were counted under high magnification (20×), with a positive microvessel defined as an endothelial cell or endothelial cell cluster that was clearly separated from adjacent microvessels, tumor cells, and other connective tissue elements.

### Statistical analysis

All statistical tests were two-sided, and the statistical analysis was performed using the GraphPad Prism version 5.0 program (GraphPad Software, CA, USA). Statistical significance was defined as *p* < 0.05, and ns = non-significant. The Student *t*-test was used to compare the mean values between two groups. Data are presented as mean values with standard deviations unless otherwise noted.

## SUPPLEMENTARY MATERIAL FIGURES


