# Supplementary material for: miR-620 promotes tumor radioresistance by targeting 15-hydroxyprostaglandin dehydrogenase (HPGD)
Source: Oncotarget. 2015 Jun 4;6(26):22439–51. doi: 10.18632/oncotarget.4210 (PMC4673174; doi:10.18632/oncotarget.4210)
Supplement: Supplementary file 1 [file oncotarget-06-22439-s001.pdf]

SUPPLEMENTARY FIGURES

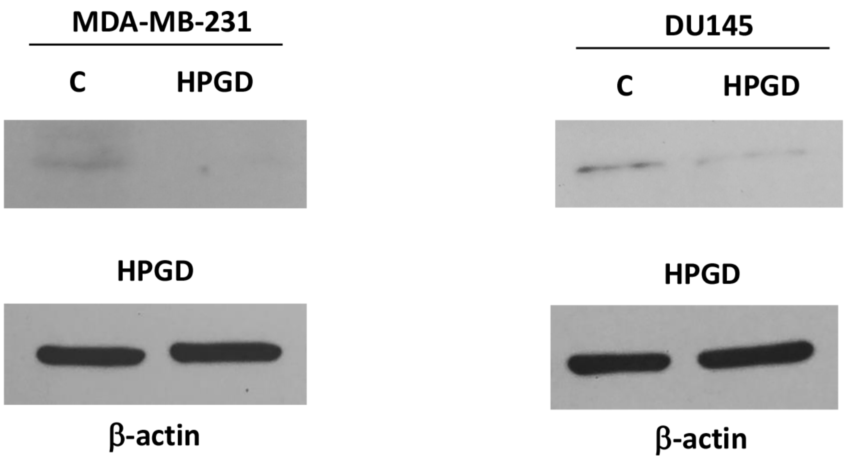

Supplementary Figure 1: HPGD protein is decreased in MDA-MB-231 and DU145 cells transfected with HPGD siRNA. Representative western blot for HPGD in whole cell lysates from MDA-MB-231 and DU145 cells transiently transfected with control or HPGD siRNA.

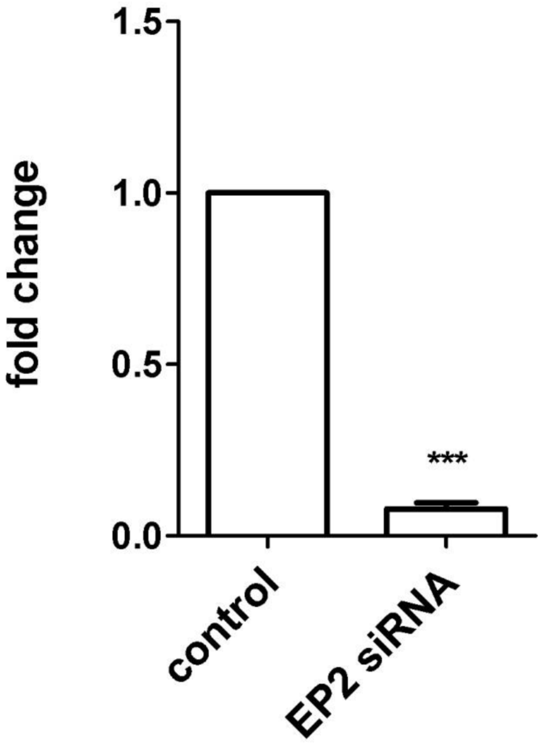

Supplementary Figure 2: Significant knockdown of EP2 expression with siRNA. MDA-MB-231 cells were transiently transfected with control or EP2 siRNA, and 24 hours later, total RNA was extracted and qRTPCR performed. Mean, standard deviations and statistical significance are denoted; ***$p < 0.001$; $n = 3$ independent experiments.
